# Books

**Published:** 1866-10

**Authors:** 


### BOOKS.

We presume it is known to many of our readers that the large and splendid book establishment of R. W. Carroll & Co. of this city was entirely consumed in the conflagration which destroyed Pike's Opera House, in April last. The loss was very great—seemingly enough to crush men of ordinary ability. But we are glad to note the fact that the establishment is again under full headway, with as full and fine a stock of all that pertains to the book trade, as is any where to be found.

The thorough resuscitation of so large an establishment, in so short a time, gives evidence of a business energy and perseverance, that no ordinary calamity can thwart. We would say to our dental and medical brethren, that they will, at all times, find a full and complete assortment of their respective professional works in this house.

Every condition and circumstance requisite to render a business transaction pleasant and satisfactory, will, at all times, be found here.

